# Spinal dorsal horn IGF1 mediates the preventive effect of electroacupuncture on cisplatin-induced peripheral neuropathy via neuronal IGF1R in mice

**DOI:** 10.1186/s13020-025-01256-1

**Published:** 2025-11-24

**Authors:** Chieh-Ru Fu, Xiao-Chen Li, Ya-Chen Yang, Hui Chen, Ruo-Fan Zhang, Yu-Xia Chu, Yan-Qing Wang, Qi-Liang Mao-Ying

**Affiliations:** 1 Department of Integrative Medicine and Neurobiology, School of Basic Medical Science, Institute of Acupuncture Research, Institutes of Integrative Medicine, Shanghai Medical College, Shanghai Key Laboratory of Acupuncture Mechanism and Acupoint Function, Fudan University, Shanghai, 200032 People’s Republic of China; 2 Shanghai Key Laboratory of Acupuncture Mechanism and Acupoint Function, Fudan University, Shanghai, 201203 People’s Republic of China; 3 State Key Laboratory of Medical Neurobiology and MOE Frontiers Center for Brain Science, Institutes of Brain Science, Shanghai Medical College, Fudan University, Shanghai, 200032 People’s Republic of China

**Keywords:** CIPN (Chemotherapy-induced peripheral neuropathy), IGF1 (Insulin-like growth factor 1), Neuron, Microglia activation, Neuroinflammation, Electroacupuncture

## Abstract

**Background:**

Our previous study demonstrated that neuronal G protein-coupled receptor kinase (GRK2) upregulation alleviated chemotherapy-induced peripheral neuropathy (CIPN) in mice, which was characterized by numbness and pain in distal hind limbs. The neuronal GRK2 was identified as a mediator of electroacupuncture (EA) effects on CIPN. Given that spinal insulin-like growth factor 1 (IGF1), a known inducer of GRK2 in the peripheral neurons, decreases after oxaliplatin treatment in mice, this study is designed to investigate whether spinal IGF1 contributes to EA-mediated prevention of cisplatin-induced peripheral neuropathy via neuronal IGF1 receptor (IGF1R).

**Methods:**

A total of 133 male C57BL/6 J mice were included in this study and randomly assigned to different experimental groups. The level of *Igf1* mRNA was detected by Real-time PCR, the p-IGF1R protein level by Western blot, after EA treatment in cisplatin-treated mice. The cellular distribution of p-IGF1R in the spinal dorsal horn was observed by immunofluorescent staining. To study the role of neuronal IGF1R in EA preventing cisplatin-induced mechanical allodynia, sensory deficit, and microglia activation and neuroinflammation in the spinal cord of mice, the neuronal IGF1R was downregulated by intraspinal injection of an AAV vector delivering IGF1R shRNA with hSyn promotor (AAV-shIGF1R). Finally, the regulatory effect of EA on spinal GRK2 was assessed by Western blot in AAV-shIGF1R mice.

**Results:**

Cisplatin treatment induced mechanical allodynia, sensory deficit, and a decrease of p-IGF1R in the spinal dorsal horn of mice. Immunofluorescence showed that p-IGF1R was localized within neurons (~ 82%), a small mount of microglia (~ 12%) and astrocytes (~ 4%). Cisplatin decreased NeuN^+^p-IGF1R^+^ neurons in the spinal dorsal horn. EA treatment significantly alleviated cisplatin-induced mechanical allodynia, sensory deficit, and significantly increased the *Igf1* mRNA and p-IGF1R level in the spinal cord. Neuronal IGF1R downregulation in the spinal dorsal horn significantly attenuated the preventive effect of EA on cisplatin-induced mechanical allodynia, sensory deficit, and spinal microglial activation and neuroinflammation in mice. Furthermore, neuronal IGF1R downregulation decreased the spinal GRK2 in cisplatin-treated mice after EA treatment. These findings suggest EA significantly alleviated CIPN symptoms by enhancing IGF1/IGF1R signaling and reducing microglial activation and neuroinflammation.

**Conclusion:**

Spinal dorsal horn IGF1 contributes to the preventive effect of EA treatment against cisplatin-induced peripheral neuropathy through neuronal IGF1R signaling in mice. The enhanced neuronal IGF1/IGF1R signaling in the spinal cord presents a potential strategy for CIPN prevention.

## Background

Chemotherapy-induced peripheral neuropathy (CIPN), characterized by debilitating sensory abnormalities in a stocking-glove distribution (e.g., numbness, persistent pain, and intraepidermal nerve fibers (IENFs) loss in the distal extremities), often persists long or even becomes irreversible [1]. Unfortunately, almost no drug has demonstrated consistent efficacy in preventing CIPN in clinics [2]. Current guidelines from American Society of Clinical Oncology (ASCO) only moderately recommend duloxetine for managing painful CIPN [3]. While duloxetine and similar agents may provide analgesia, their therapeutic utility remains limited as they fail to reverse the course of disease [2, 4].

Acupuncture, as a key therapeutic approach in traditional Chinese medicine, has demonstrated efficacy in alleviating CIPN symptoms both in clinical and animal researches [5–7]. Moreover, electroacupuncture (EA) at the Zusanli (ST36) acupoints can attenuate chemotherapy-induced mechanical hypersensitivity in rats [8]. Our earlier studies further indicated that EA treatments can prevent cisplatin-induced CIPN symptoms and downregulation of G protein-coupled receptor kinase 2 (GRK2) in the spinal cord dorsal horn of mice [9, 10]. Crucially, neuronal GRK2 downregulation in the spinal dorsal horn abolishes the preventive effects of EA in cisplatin-treated mice, directly implicating spinal neuronal GRK2 as a mediator of the EA-driven CIPN prevention [9].

Insulin-like growth factor 1 (IGF1), widely distributed in the nervous system [11–14], exerts its biological effects through binding to the IGF1 receptor (IGF1R). Notably, the IGF1 level in the spinal cord decreases in oxaliplatin-treated mice [15], while peripheral IGF1 administration elevates the GRK2 level in the dorsal root ganglia in mice [16]. However, the role of spinal dorsal horn IGF1/IGF1R in EA alleviating cisplatin-induced CIPN remains to be elucidated.

Therefore, we propose that spinal dorsal horn IGF1 mediates the preventive effect of EA against cisplatin-induced CIPN via neuronal IGF1R in mice. Our research has demonstrated that EA treatment upregulates IGF1/IGF1R in the spinal dorsal horn of cisplatin-treated mice. Through AAV-mediated genetic manipulation, we found that neuronal IGF1R downregulation in the spinal dorsal horn significantly attenuated the protective effects of EA against cisplatin-induced mechanical allodynia, sensory deficit, and microglia activation and neuroinflammation in the spinal dorsal horn. Moreover, neuronal IGF1R downregulation decreased the GRK2 level in the spinal dorsal horn after EA treatment in cisplatin-treated mice. Our findings suggest that spinal IGF1/IGF1R–mediated regulation of GRK2 is a key mechanism underlying the preventive effects of EA against cisplatin-induced CIPN in mice.

## Methods

### Animals

Male adult C57BL/6 J mice (8–10 weeks old) were obtained from the Shanghai Laboratory Animal Center, Chinese Academy of Sciences. Animals were housed under controlled conditions (22 ± 0.5 °C, 12 h light/12 h dark cycle) with free food and water ad libitum. The mice were adapted to the experimental environment for at least two weeks before any manipulations. The experiment was designed as a single study, approved by local ethical committee at School of Basic Medical Sciences, Fudan University, People’s Republic of China (Agreement No.20220228–103). Mice were randomly assigned to different experimental groups, and the group allocation was concealed by encoded labels.

### Induction of CIPN model

The CIPN model was established following our established protocols [9, 17]. Briefly, the mice received intraperitoneal (i.p.) injections of cisplatin (2.3 mg/kg, Sigma Aldrich) for 5 consecutive days, repeated in two cycles separated by a 5-day interval. The control mice received i.p. injections of equivalent volume of sterile saline as the vehicle control.

### Intraspinal adeno-associated virus injections

To achieve neuron-specific IGF1R downregulation in the spinal cord, a mixture of adeno-associated virus (AAV) AAV2/8-hSyn-DIO-EGFP-5'miR30-shRNA(IGF1R)-3'miR30-WPREs (5.86 × 10^12^GC/600 nl, BrainVTA, China) with AAV2/8-hSyn-creERT2-WPRE-hGHpA (5.53 × 10^12^GC/600 nl, BrainVTA, China) (2:1 volume) was stereotaxically injected into the spinal dorsal horn of mice three weeks before cisplatin injection using our established protocol [9]. The control mice received AAV2/8-hSyn-DIO-EGFP-5'miR30-shRNA(scramble)-3'miR30-WPREs (5.92 × 10^12^GC/300 nl, BrainVTA, China). In brief, after exposure of the spinal dorsal surface between T13 and L1 vertebra, a glass capillary (outer diameter of 1.14 mm, inner diameter of 0.53 mm) connected to a micro-injection pump (Nanoliter 2010, World Precision Instruments) was stereotaxically inserted into the spinal dorsal horn at a depth of 200–250 μm. The virus suspension was infused at 60 nl/min, and the capillary was left for extra 10 min before pulled out. Finally, the wound was closed, and the mice were kept on a heating pad until waking up from anesthesia. Three weeks after the virus administration, tamoxifen (1 mg/100 μl) was i.p. injected for 5 consecutive days to induce Cre activity.

### von Frey test

Following our established protocols [9, 18], a series of von Frey hairs (0.02, 0.04, 0.07, 0.16, 0.4, 0.6, 1.0 and 1.4 g) (Stoelting, USA) was used to measure mechanical withdrawal threshold by using Dixon’s up-and-down method. In brief, the mice were adapted in specially designed plastic chambers at least 30 min before the testing. The testing started with 0.16 g von Frey hair. If a negative response was observed, the hair force was increased. Otherwise, the hair force was decreased. After the first change in the response pattern, four more stimuli were observed. Finally, 50% mechanical withdrawal threshold was counted.

### Adhesive removal test

As previously described [9, 17], the sensory capability of hind paw was evaluated through adhesive removal test. In brief, a round piece of adhesive paper (3/16 inches Teeny Touch-Spots) was applied on the plantar surface of hind paws. The performance of the mice was recorded in a testing cage for 15 min. The time from applying the paper to the mice shaking or licking its hind paws for the first time was counted as the response time of adhesive paper.

### Open field test (OFT)

According to the previous study [19], the locomotor activity of mice was evaluated by open field test. In brief, the performance of mice in a white box with 50 cm × 50 cm floor and 40 cm height wall was recorded by a camera for 5 min. The total traveled distance was counted as the locomotor activity of mice.

### Rotarod test

The mice were trained twice a day on the static drum before the test session, and each session sustained for 5 min at 5 rpm (a relatively low speed). For the test session, the speed was accelerated from 4 to 40 rpm in 300 s. The mean value of falling latency was calculated by three trials in each mouse.

### EA treatment

Previous studies have demonstrated that EA treatment (10 Hz, 2 mA, 0.4 ms) can significantly alleviate chemotherapy-induced neuropathic pain in rats [8]. Our prior work also indicated that EA treatments at Zusanli (ST36) and Kunlun (BL60) with a Han’s Acupoint Nerve Stimulator (LH202, Huawei Co. Ltd., Beijing, China) at 2 Hz/100 Hz (1 mA for 15 min followed by 2 mA for 15 min) effectively prevented and mitigated cisplatin-induced peripheral neuropathy in mice [9]. Therefore, the present study employed EA treatments (10 Hz, 0.4 ms) at ST36 and BL60 to investigate its effects on cisplatin-induced peripheral neuropathy in mice. To minimize stress responses caused by electrical stimulation, a stepwise protocol of 1 mA for 15 min followed by 2 mA for 15 min was applied. Briefly, the mice were gently restrained by a specially designed holder, allowing free movement of the head and the four limbs in accordance with previous publications [9, 10]. Two pairs of acupuncture needles with a diameter of 0.16 mm, were inserted bilaterally into Zusanli acupoint (ST36, posterolateral of the knee joint, 2 mm below the fibular head, interosseous space between tibia and fibula) and Kunlun acupoint (BL60, located at the depression between the lateral ankle of the hind limb and the Achilles tendon) at depths of 3 and 2 mm. The ipsilateral Kunlun and Zusanli acupoints were connected with Master-9 pulse stimulator (A.M.P.I.). A train of square-wave pulses at 10 Hz with 0.4 ms were applied with current intensities of 1 mA and 2 mA, respectively, for a duration of 15 min consecutively. To observe the preventive effect of EA, the EA treatment was started one day before the first cisplatin injection, and ended one day after the last cisplatin injection. For sham EA treatment, the needles were inserted into Zusanli and Kunlun acupoints without electrical stimulation.

### Western blotting

After deep anesthesia with an overdose of 2.5% avertin, the L4-L6 spinal dorsal horn of mice was quickly separated and homogenized in RIPA lysis buffer. The homogenate was centrifuged at 14,000 rpm for 20 min at 4 °C, and then the supernatant was mixed with 4 × loading buffer (ratio: 3:1). The rabbit anti-p-IGF1R (1:1000, Cell Signaling catalog no.3918S), rabbit anti-IGF1R (1:1000, Cell Signaling catalog no.3918S), mouse anti-GRK2 (1:1000, Santa cruz catalog no.Sc-13143) or β-actin (1:10,000, Proteintech catalog no.HRP-66009) primary antibodies were used to evaluate the levels of p-IGF1R, IGF1R, GRK2 by Western blotting according to our previous method [17]. For electrophoresis, a volume of protein sample (20–25 μL) was carefully loaded into each well using a micropipette, and 3–5 μL of protein marker was applied to wells on both sides of the gel to serve as molecular weight references. After incubation with the secondary antibody (1:10,000, Proteintech), Western blot images were captured by an ImageQuant LAS4000 mini-image analyzer (GE Healthcare), and the levels of p-IGF1R, IGF1R, GRK2 were analyzed by ImageJ (version 2.9.0). The expression level of p-IGF1R was normalized to IGF1R. The relative expression levels of IGF1R and GRK2 were normalized to β-actin, which served as an internal loading control to correct for variations in protein loading and transfer. The values were further normalized to the saline group.

### Immunofluorescence staining

According to the previous description [9, 17], the L4-L6 spinal cord was immersed in 4% formaldehyde for 24 h, and the skin from the center of the hind paws was fixed in Zamboni’s fixative for 24 h. Then, the spinal cord and skin were transferred to 30% sucrose for dehydration until the tissue sank. The tissue was sliced into 25 μm sections. The sections were incubated in blocking solution at room temperature for 1–2 h, and then transferred to primary antibodies and incubated at 4 °C overnight on the shaking table (Table 1). Finally, the sections were incubated with Alexa Fluor 488 or Alexa Fluor 594-conjugated secondary antibodies (1: 1000, Invitrogen) at room temperature for 1–2 h. The images were captured under a confocal microscope (Olympus FV3000), and the p-IGF1R, Iba1 fluorescence intensity was quantified using ImageJ (version 2.9.0).

**Table 1:** Information of primary antibodies for immunofluorescence

Name	Information	Application
Mouse anti NeuN	Merck catalog no.MAB377	1: 400
Goat anti IBA1	Invitrogen catalog no.PA5-18,039	1: 500
Mouse anti GFAP	Cell signaling catalog no.3670S	1: 500
Rabbit anti p-IGF1R	Cell signaling catalog no.3918S	1: 400

### Real-time PCR analysis

According to the previous description [9, 17], total mRNA was extracted from L4-L6 spinal dorsal horn by Trizol reagent (Invitrogen, USA). Following the manufacturer’s instructions, reverse transcription was performed using PrimeScript™ RT reagent Kit with gDNA Eraser (Takara, Japan). Quantitative Real-time PCR analysis was conducted using SYBR^®^ Premix Ex Taq™ I (Takara, Japan) and the ABI 7300 Plus Detection System (Applied Biosystems, LA, USA). The primers utilized in this study are listed in Table 2.

**Table 2:** Primes for real-time PCR

Gene	Primer forward (5′-3′)	Primer reverse (5′-3′)
*Cd16*	5′-AGACCCAGCAACTACATCC-3′	5′-GACTTCCTCCAGTAATCCCT-3′
*Cd206*	5′-GCTTCCGTCACCCTGTATG-3′	5′-CTCCACAATCCCGAACCT-3′
*Il-4*	5′-CCATGAATGAGTCCAAGTCC-3′	5′-TGATGCTCTTTAGGCTTTCC-3′
*Il-10*	5′-GGGAAGAGAAACCAGGGAGA-3′	5′-GGGGATGACAGTAGGGGAAC-3′
*inos*	5′-TTGACGCTCGGAACTGTAG-3′	5′-GACCTGATGTTGCCATTGT-3′
*Il-1β*	5′-GTACAAGGAGAACCAAGCAA-3′	5′-CCGTCTTTCATTACACAGGA-3′
*Tnf-α*	5′-ACTCTGACCCCTTTACTCTG-3′	5′-GAGCCATAATCCCCTTTCTA-3′
*Il-6*	5′-CCAATGCTCTCCTAACAGAT-3′	5′-TGTCCACAAACTGATATGCT-3′
*Gapdh*	5′-AAATGGTGAAGGTCGGTGTG-3′	5′-AGGTCAATGAAGGGGTCGTT-3′

### Statistical analysis

All behavioral testing and histological quantification were performed under blinded conditions, with investigators unaware of treatment allocation until completion of analysis. All data in the present study are presented as mean ± standard error of mean (SEM). The statistical significance of differences was analyzed with Student’s T-test, one-way or two-way analysis of variance (ANOVA) following Turkey post-test. P < 0.05 was the threshold of significance.

## Result

### Cisplatin treatment decreased p-IGF1R in the neuron of spinal dorsal horn

**Fig. 1:**
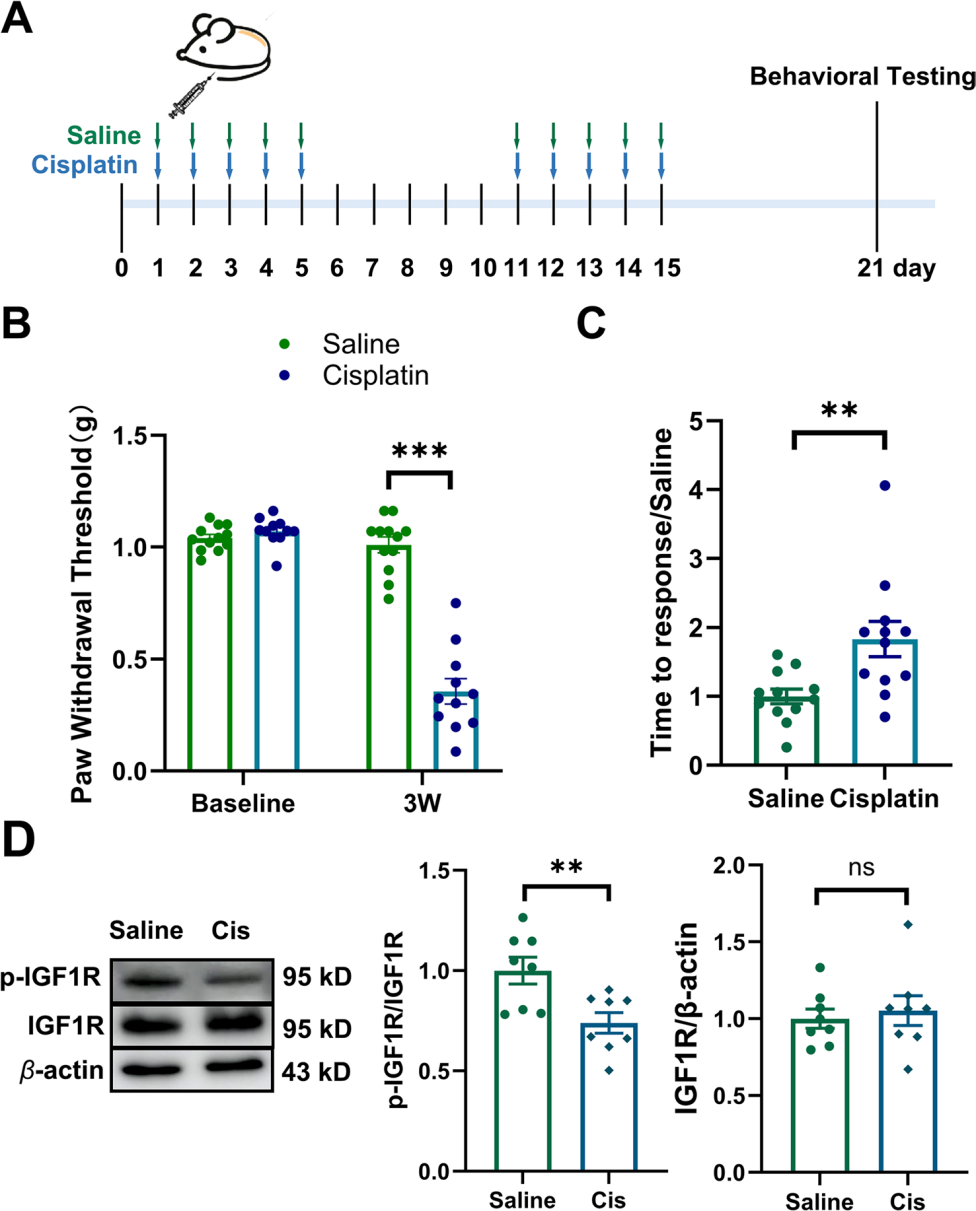
Cisplatin treatment induced mechanical allodynia and sensory deficit in mice. **A** Schedule of cisplatin treatment and behavioral test. **B** Repeated cisplatin treatments decreased the paw withdrawal threshold in von Frey test. Values are represented as mean ± SEM. n = 12 for saline group, 11 for cisplatin group. The statistical method is repeated measures ANOVA followed by Tukey test for multiple comparisons. For post hoc analysis, ***p < 0.001, η^2^ = 0.24, 95% CI [0.24, 0.38]. **C** Repeated cisplatin treatments increased response time in adhesive removal test. Values are represented as mean ± SEM. n = 12 for saline group and cisplatin group. The statistical method is unpaired t-test. **p < 0.01, η^2^ = 0.29, 95% CI [0.26, 1.40]. **D** Western blot analysis revealed a decrease of p-IGF1R, but no change of IGF1R in the spinal dorsal horn by downregulating neuronal IGF1R after cisplatin treatment. Results are normalized to β-actin. n = 8 for each group. Values are mean ± SEM. The statistical method is unpaired t-test. **p < 0.01, η^2^ = 0.40, 95% CI [− 0.43, − 0.08]

**Fig. 2:**
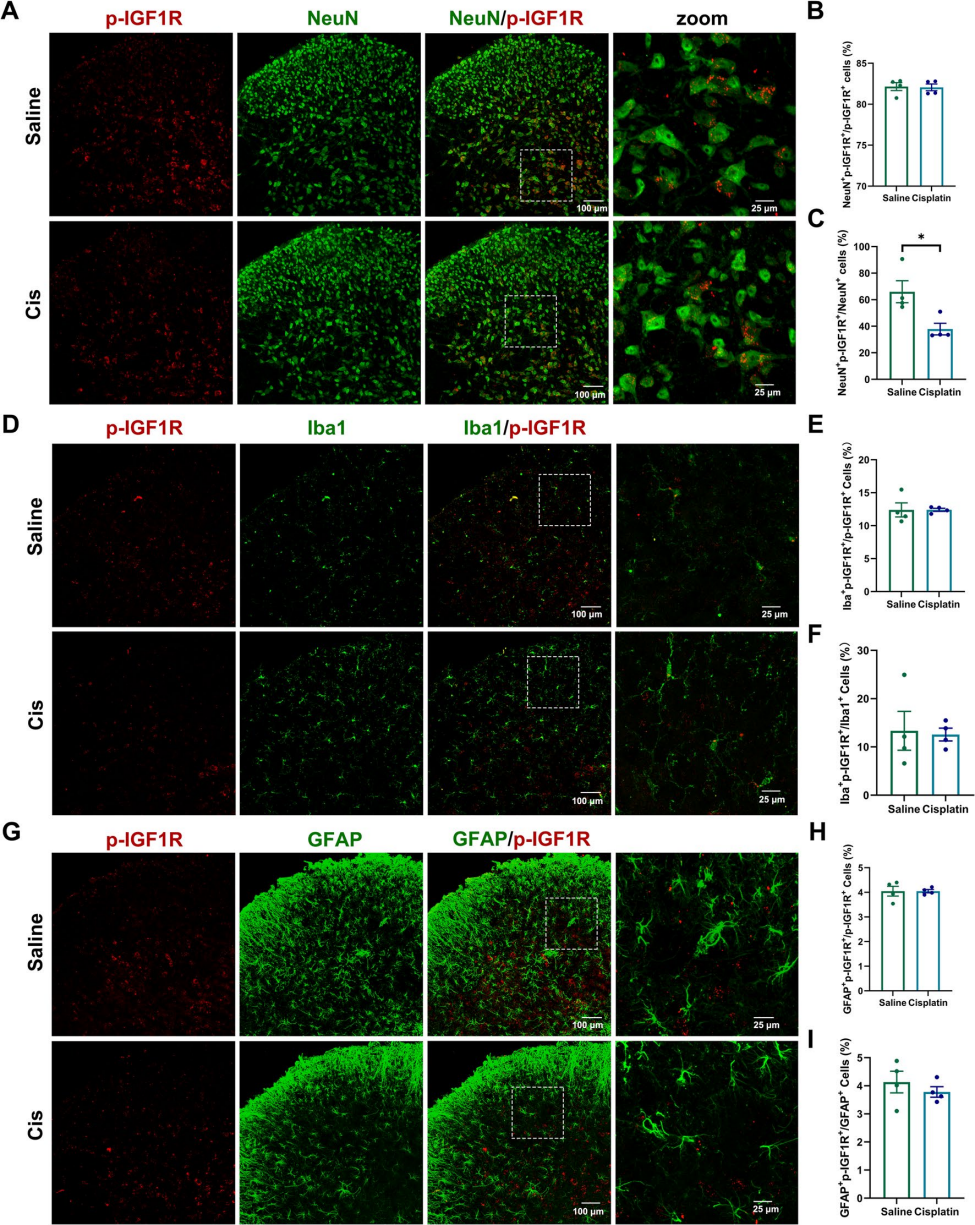
The cellular distribution of p-IGF1R in the spinal dorsal horn. **A** The representative images of p-IGF1R co-localized with NeuN in the spinal dorsal horn of saline- or cisplatin-treated mice. **B**,** C** The percentage of NeuN^+^p-IGF1R^+^ cells in p-IGF1R^+^ and NeuN^+^ cells. Values are represented as mean ± SEM. n = 4 for saline group and cisplatin group. The statistical method is unpaired t-test. *p < 0.01, η^2^ = 0.59, 95% CI [− 51.1, − 5.14]. **D** The representative images of p-IGF1R co-localized with Iba1 in the spinal dorsal horn of saline- or cisplatin-treated mice. **E**–**F** The percentage of Iba1^+^p-IGF1R^+^ cells in p-IGF1R^+^ and Iba1^+^ cells. Values are represented as mean ± SEM. n = 4 for saline group and cisplatin group. The statistical method is unpaired t-test. **G** The representative images of p-IGF1R co-localized with GFAP in the spinal dorsal horn of saline- or cisplatin-treated mice. **H**–**I** The percentage of GFAP^+^p-IGF1R^+^ cells in p-IGF1R^+^ cells and GFAP^+^ cells. Values are represented as mean ± SEM. n = 4 for saline group and cisplatin group. The statistical method is unpaired t-test

It has been reported that oxaliplatin reduces IGF1 level in the spinal cord, which may contribute to oxaliplatin-induced peripheral neuropathy in mice [15]. IGF1 is known to exert its biological role in regulating receptor autophosphorylation and the activity of receptor through binding to the extracellular IGF1R [20, 21]. In this study, repeated cisplatin treatment decreased the paw withdrawal threshold in response to von Frey stimulation and prolonged the response time to adhesive paper in mice (Fig. 1A–C). Western blotting revealed that cisplatin reduced p-IGF1R protein level in the spinal dorsal horn of mice (Fig. 1D). Immunofluorescent staining demonstrated that p-IGF1R was predominantly localized in the spinal cord neurons (~ 82%) (Fig. 2A, B). Cisplatin treatment significantly reduced the proportion of NeuN^+^p-IGF1R^+^ cells relative to total NeuN^+^ cells (Fig. 2C). Furthermore, only minimal p-IGF1R localization was observed in astrocyte (~ 4%) and microglia (~ 12%) (Fig. 2D–I).

### EA increased *Igf1* mRNA and p-IGF1R protein in the spinal dorsal horn of cisplatin-treated mice

**Fig. 3:**
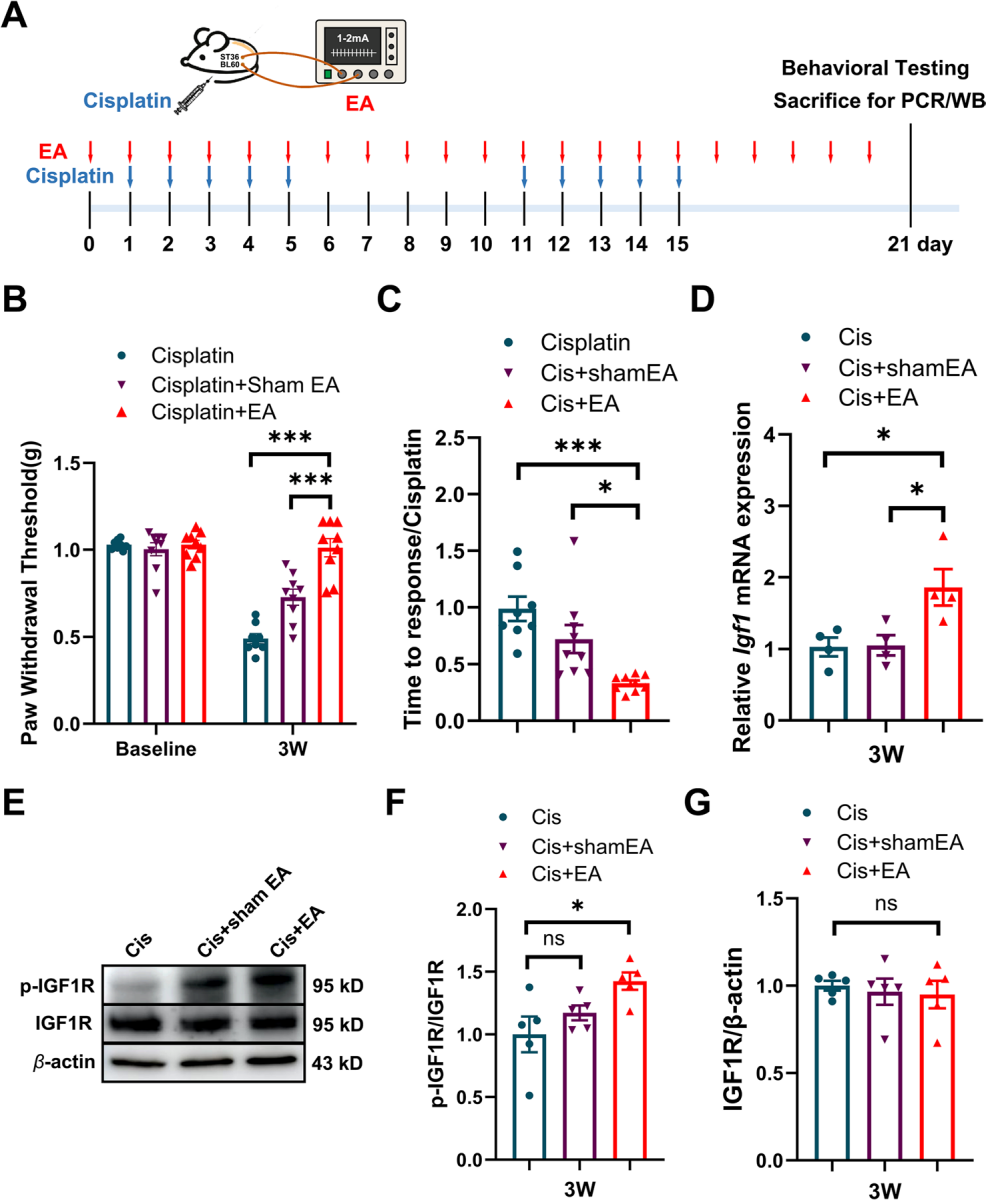
The effect of EA treatment on cisplatin-treated mice. **A** Schedule of cisplatin and EA treatment. **B** EA treatment increased the paw withdrawal threshold of cisplatin-treated mice in von Frey test. n = 8–9. Values are mean ± SEM. The statistical method is two-way ANOVA followed by Tukey test for multiple comparisons. For post hoc analysis, ***p < 0.001, η^2^ = 0.22, 95% CI [0.21, 0.34]. **C** EA treatment decreased the response time of cisplatin-treated mice in adhesive removal test. n = 8–9. Values are mean ± SEM. The statistical method is one-way ANOVA followed by Tukey test for multiple comparisons. For post hoc analysis, *p < 0.05, ***p < 0.001, η^2^ = 0.5, 95% CI [0.30, 1.00]. **D** Real-time PCR analysis of *Igf1* mRNA in the spinal dorsal horn of cisplatin-treated mice after EA treatment. n = 4 for cisplatin group, cisplatin + sham EA group and cisplatin + EA group. Values are mean ± SEM. The statistical method is one-way ANOVA followed by Tukey test for multiple comparisons. For post hoc analysis, *p < 0.05, η^2^ = 0.59, 95% CI [− 1.59, − 0.06]. **E** The representative image of p-IGF1R and IGF1R protein in the spinal dorsal horn of cisplatin-treated mice after EA treatment. **F**–**G** The analysis of protein level of p-IGF1R and IGF1R in the spinal dorsal horn of cisplatin-treated mice after EA treatment. Results are normalized to β-actin and shown as ratios to cisplatin-treated mice. n = 5 for cisplatin group, cisplatin + sham EA group and cisplatin + EA group. Values are mean ± SEM. The statistical method is one-way ANOVA followed by Tukey test for multiple comparisons. For post hoc analysis, *p < 0.05, η^2^ = 0.44, 95% CI [− 0.80, − 0.04]

Preventive EA treatment has been demonstrated to be effective in preventing cisplatin-induced CIPN in mice [9, 10]. In this study, EA treatment, started one day before the 1st cisplatin treatment, was applied once a day until the end of the experiment (Fig. 3A). Continuous EA treatment significantly increased the paw withdrawal threshold in von Frey test, and significantly decreased the response time in adhesive removal test (Fig. 3B, C). qRT-PCR analysis revealed that EA treatment significantly increased the *Igf1* mRNA level in the spinal cord of cisplatin-treated mice, as compared to cisplatin-treated mice or cisplatin + sham EA mice (Fig. 3D). Western blotting further demonstrated that preventive EA upregulated the protein level of p-IGF1R, but not total IGF1R, in the spinal cord of cisplatin-treated mice (Fig. 3E–G). These results suggest that EA may exert its preventive effect on CIPN by enhancing IGF1/p-IGF1R signaling in the spinal dorsal horn.

### Spinal neuronal IGF1R contributed to the EA preventing cisplatin-induced mechanical allodynia and sensory deficit in mice

**Fig. 4:**
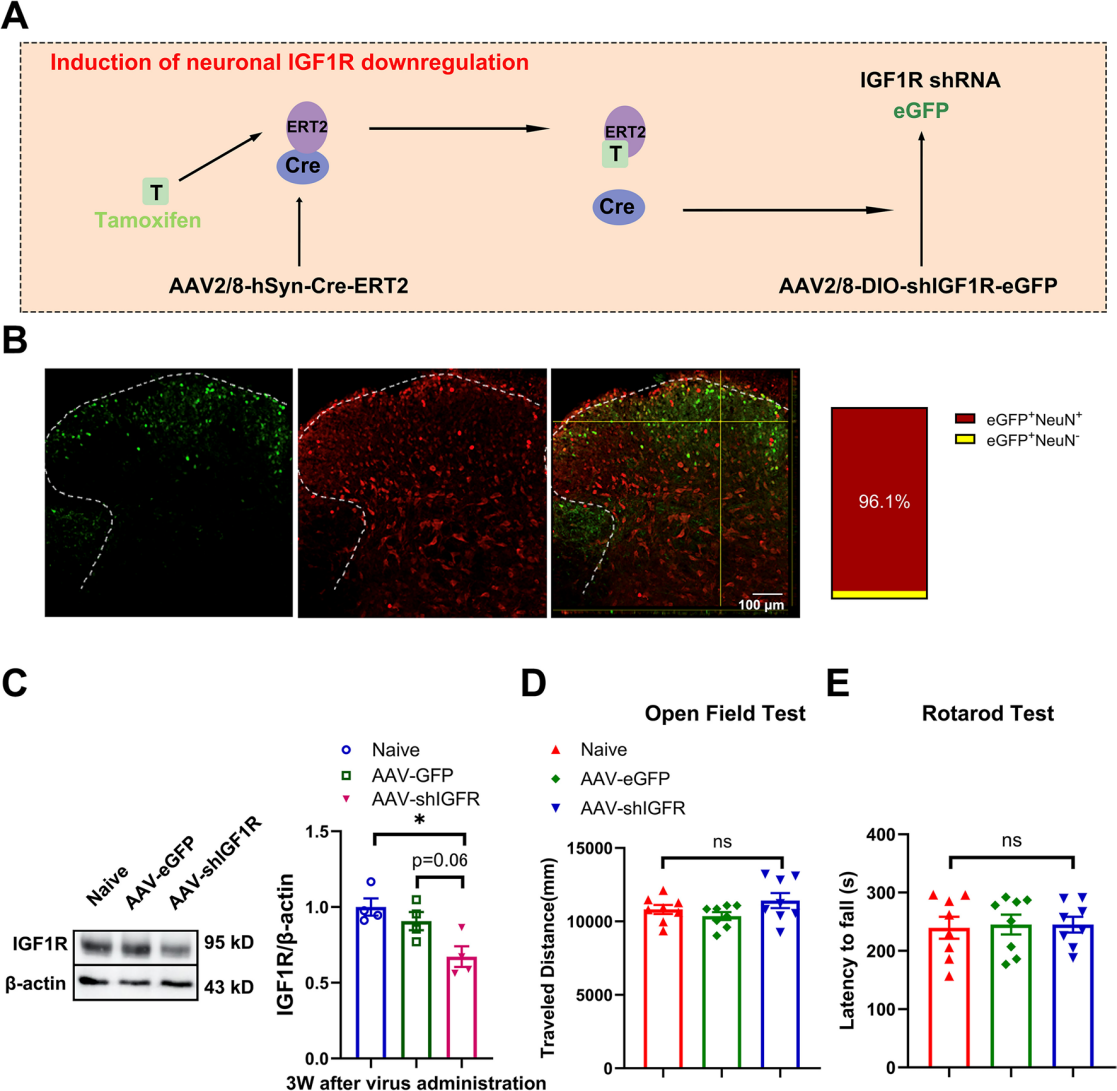
Downregulation of neuronal IGF1R in the spinal dorsal horn after intraspinal injection of adeno-associated virus. **A** The working mode of neuronal IGF1R downregulated through intraspinal injection of adeno-associated virus. **B** The immunofluorescent results of AAV-expressed eGFP (green) co-localizes with NeuN (red) in the spinal dorsal horn. White arrow: representative neuron (red) expressed eGFP (green). Scale bar: 100 μm. **C** Western blot analysis revealed a decrease of IGF1R in the spinal dorsal horn after intraspinal injection of adeno-associated virus. Results are normalized to β-actin. n = 4 for each group. Values are mean ± SEM. The statistical method is one-way ANOVA followed by Tukey test for multiple comparisons. For post hoc analysis, *p < 0.05, η^2^ = 0.62, 95% CI [0.08, 0.57]. **D**–**E** Effect of neuronal IGF1R downregulation on locomotor performance in open field test and rotarod test. n = 8 for each group. Values are mean ± SEM. The statistical method is one-way ANOVA

**Fig. 5:**
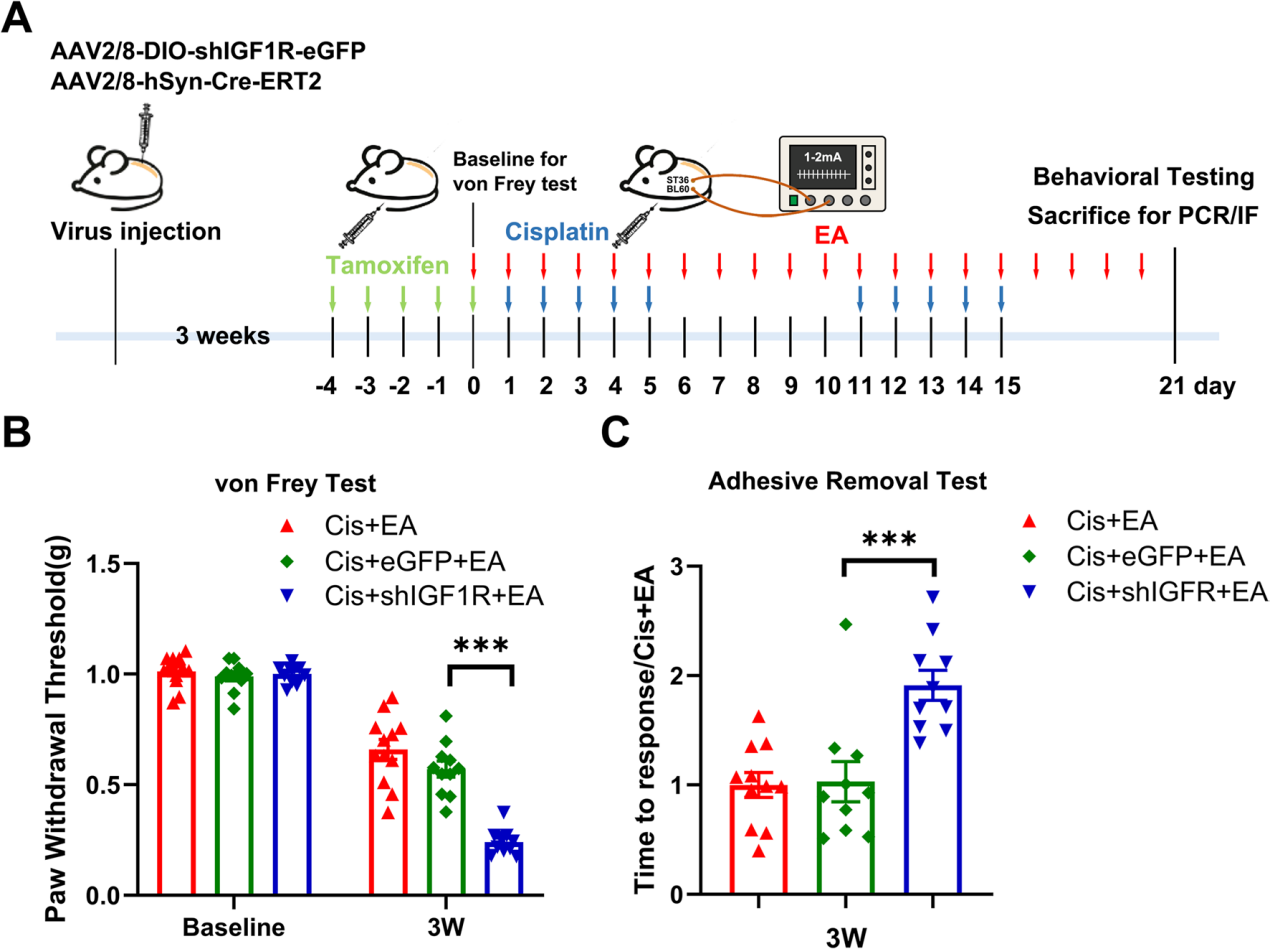
Downregulation of neuronal IGF1R in the spinal dorsal horn inhibited the preventive effect of EA on cisplatin-induced mechanical allodynia and sensory deficits. **A** Schedule of neuronal IGF1R knockdown and EA treatment in cisplatin-treated mice. Baseline was defined as day 0, corresponding to the time point after the final tamoxifen injection when the viral construct was activated. Electroacupuncture treatment was initiated after baseline behavioral test, one day prior to cisplatin injection. **B** Downregulation of neuronal IGF1R decreased paw withdrawal threshold following EA treatment. n = 10–12. Values are mean ± SEM. The statistical method is two-way repeated measures ANOVA followed by Tukey test for multiple comparisons. For post hoc analysis, ***p < 0.001, η^2^ = 0.93, 95% CI [0.22, 0.43] **C** Downregulation of neuronal IGF1R extended the response time in mice following EA treatment. n = 10–11. Values are mean ± SEM. The statistical method is one-way ANOVA followed by Tukey test for multiple comparisons. For post hoc analysis, ***p < 0.001, η^2^ = 0.47, 95% CI [− 1.41, 0.34]

To assess the role of neuronal IGF1R in mediating the preventive effects of EA on cisplatin-induced CIPN, we designed a tamoxifen-inducible system using two AAV vectors: (1) AAV2/8-DIO-shIGF1R-eGFP (AAV-shIGF1R) to deliver IGF1R-targeting shRNA, and (2) AAV2/8-hSyn-Cre-ERT2 under a neuron-specific promoter to enable spinal neuronal IGF1R knockdown in mice (Fig. 4A). Immunofluorescence analysis confirmed that AAV-shIGF1R (green) was localized within NeuN^+^ neurons (red) in the spinal dorsal horn three weeks after intraspinal injection of AAV vectors (Fig. 4B). A decrease of IGF1R level in the spinal dorsal horn was determined after AAV-shIGF1R by Western blotting analysis (Fig. 4C). In addition, neuronal IGF1R downregulation in the spinal cord had no significant impact on locomotor performance in open field or Rotarod tests (Fig. 4D, E). To verify the role of neuronal IGF1R in EA-mediated alleviation of cisplatin-induced CIPN, we employed an EA-treated mouse CIPN model using an empty eGFP viral vector as a control. In the mice with spinal neuronal IGF1R downregulation (cis + shIGF1R + EA), a decrease of mechanical paw withdrawal threshold in von Frey test, and an increase of response times in adhesive removal test were observed after EA treatment in cisplatin-treated mice compared to cisplatin + eGFP + EA (Fig. 5). These demonstrate that spinal neuronal IGF1R downregulation inhibits the protective effects of EA against mechanical allodynia and sensory deficits in cisplatin-treated mice.

### Spinal neuronal IGF1R contributed to the EA preventing cisplatin-induced microglial activation and neuroinflammation in mice

**Fig. 6:**
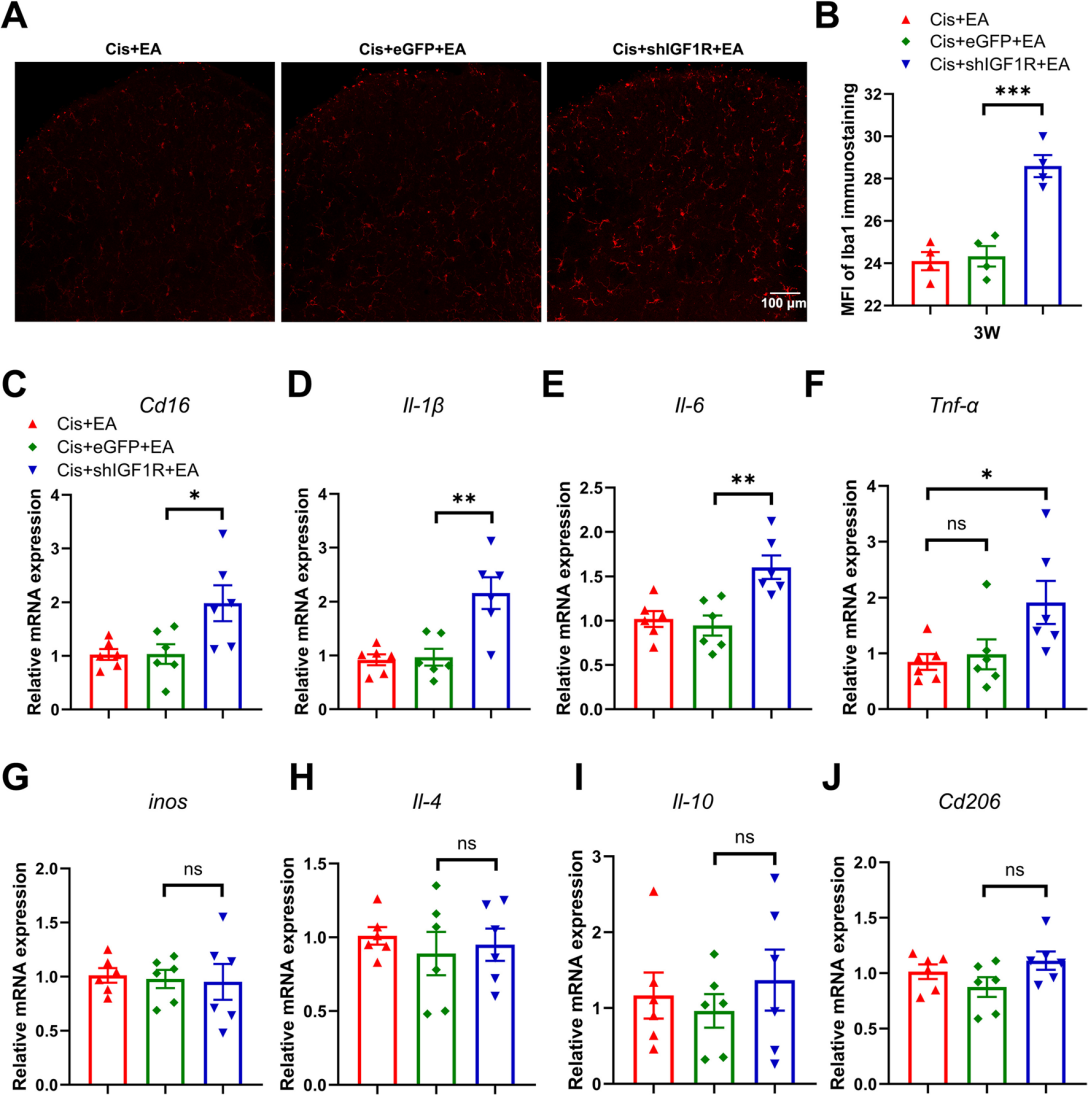
Downregulation of neuronal IGF1R in the spinal dorsal horn inhibited the preventive effect of EA on spinal microglia activation and neuroinflammation. **A** Immunofluorescence staining of Iba1 in cisplatin-treated mice after virus administration and EA treatment. Scale bars: 100 μm. **B** The analysis of Iba1 immunoreactivity. n = 4 for each group. Values are mean ± SEM. The statistical method is one-way ANOVA followed by Tukey test for multiple comparisons. For post hoc analysis, ***p < 0.001, η^2^ = 0.86, 95% CI [− 6.25, − 2.27]. **C**–**J** Real-time PCR analysis of *Cd16*, *Il-1β, Il-6, Tnf-α, inos, Il-4*, *Il-10, Cd206*. Results are normalized to *Gapdh*. n = 6 for each group. Values are mean ± SEM. The statistical method is one-way ANOVA followed by Tukey test for multiple comparisons. For post hoc analysis, *p < 0.05, **p < 0.01, *Cd16:*η^2^ = 0.43, 95% CI [− 1.81, − 0.07]; *Il-1β:* η^2^ = 0.61, 95% CI [− 1.96, − 0.42]*; Il-6:* η^2^ = 0.57, 95% CI [− 1.07, − 0.24]*; Tnf-α::*η^2^ = 0.35, 95% CI [− 2.11, − 0.02]

Our previous studies have demonstrated that EA treatment suppresses microglial activation and neuroinflammation in the spinal dorsal horn in cisplatin-treated mice [9, 10]. In this study, immunofluorescence analysis revealed significantly elevated Iba1^+^ signal intensity in the cisplatin + shIGF1R + EA mice as compared to cisplatin + eGFP + EA mice (Fig. 6A, B). qRT-PCR further indicated upregulated mRNA levels of *Cd16* (an M1 microglia marker), *Il-1β* and *Il-6* in the spinal dorsal horn of cisplatin + shIGF1R + EA mice, whereas an increase trend of *Tnf-α* and no changes of *inos*, *Il-4*, *Il-10*, or *Cd206* (an M2 microglia marker) were observed as compared to cisplatin + eGFP + EA mice or cisplatin + EA mice (Fig. 6C–J). These results suggest that spinal neuronal IGF1R downregulation attenuates the protective effects of EA against cisplatin-induced microglial activation and neuroinflammation. Collectively, our data indicate that the EA-mediated upregulation of spinal IGF1 may contribute to its prevention of cisplatin-induced CIPN through neuronal IGF1R signaling in mice.

### Spinal neuronal IGF1R contributed to the EA effect in regulating GRK2 in the spinal dorsal horn of mice

**Fig. 7:**
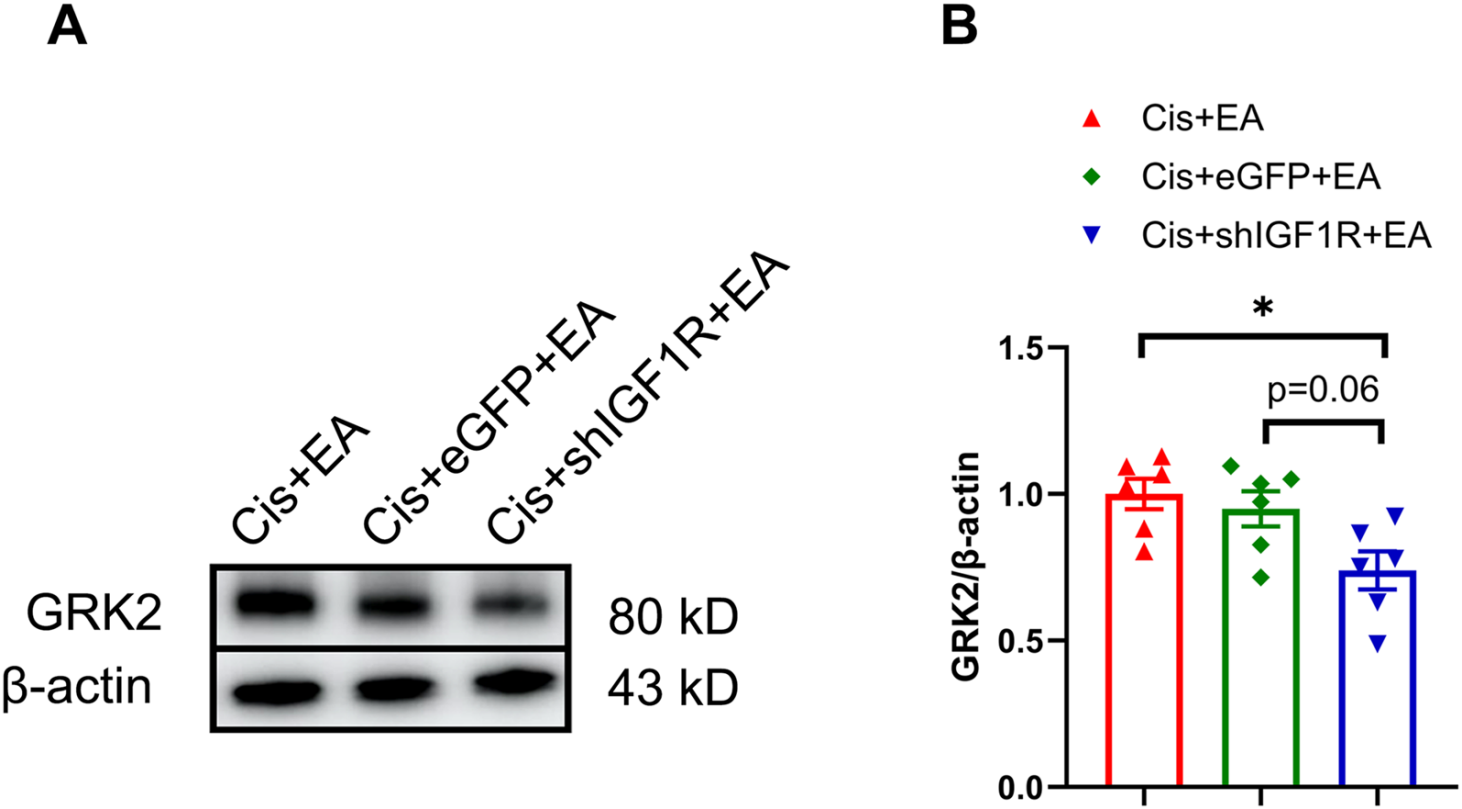
Western blot analysis revealed a decrease of GRK2 in the spinal dorsal horn by downregulating neuronal IGF1R after electroacupuncture treatment. Results are normalized to β-actin. n = 6 for each group. Values are mean ± SEM. The statistical method is one-way ANOVA followed by Tukey test for multiple comparisons. For post hoc analysis, *p < 0.05, η^2^ = 0.41, 95% CI [0.04, 0.47]

**Fig. 8:**
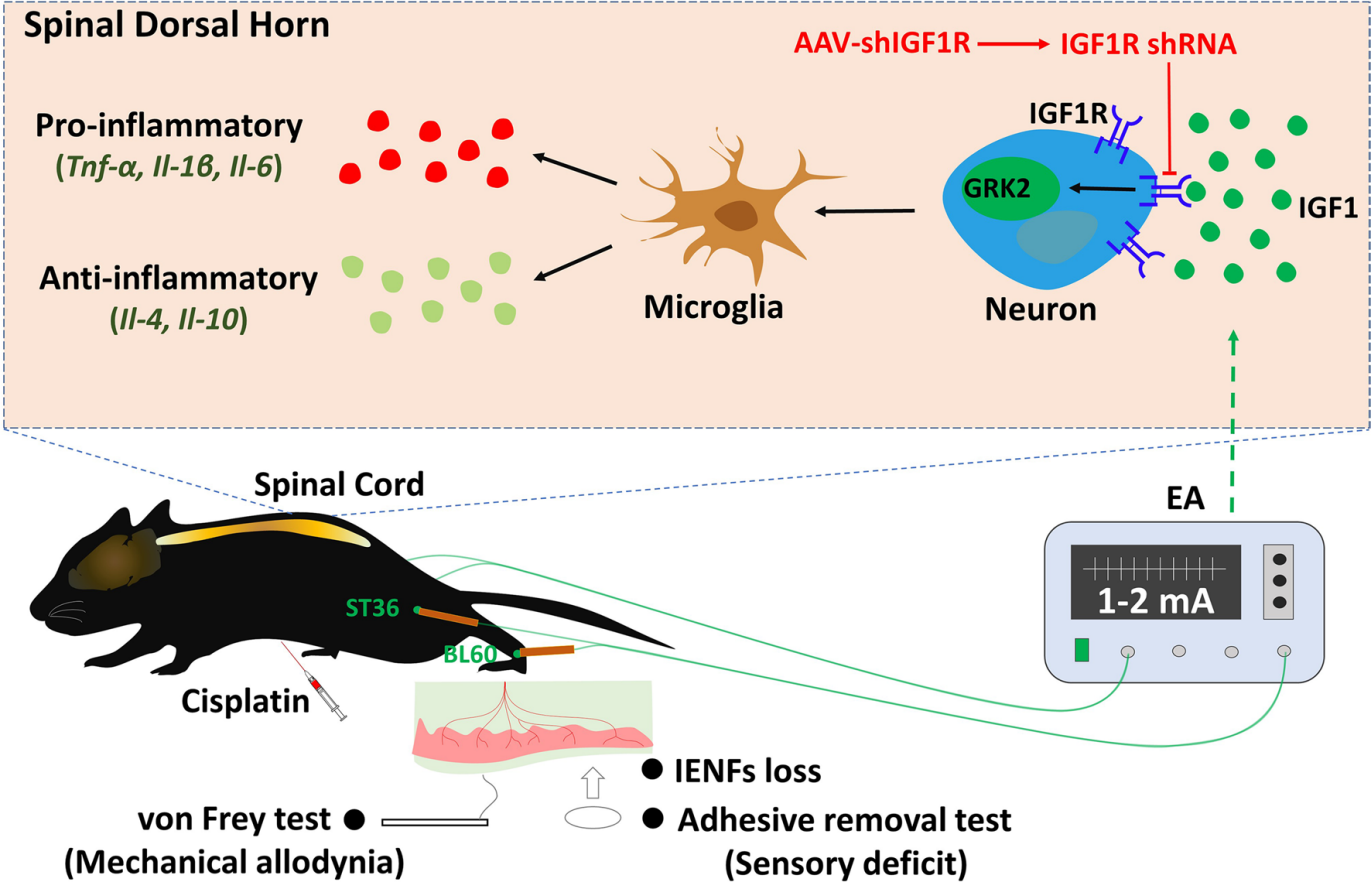
Schematic representation of our working model. EA upregulated IGF1, which regulated neuronal GRK2 through IGF1R, and then microglia activation and neuroinflammation in the spinal dorsal horn, finally contributing to the preventive process on cisplatin-induced CIPN by EA. This preventive process was inhibited by silencing the neuronal IGF1R in cisplatin-treated mice

EA was shown to upregulate spinal GRK2 in cisplatin-treated mice, and neuronal GRK2 in the spinal dorsal horn has been implicated in mediating EA’s protective effects against cisplatin-induced CIPN [9]. In this study, Western blot analysis revealed that neuronal IGF1R downregulation decreased the GRK2 level in the spinal dorsal horn in cisplatin + EA-treated mice (Fig. 7). Together, our data indicate that spinal IGF1 regulates neuronal GRK2 via IGF1R, which likely underlies the mechanism by which EA prevents cisplatin-induced CIPN (Fig. 8).

## Discussion

In the present study, EA treatment significantly increased the *Igf1* mRNA and p-IGF1R, which was mainly distributed in the neurons of spinal dorsal horn, in cisplatin-treated mice. Our previous research showed that EA could prevent cisplatin-induced mechanical allodynia, sensory deficit, and microglia activation and neuroinflammation in the spinal dorsal horn induced by cisplatin in mice [9, 10]. The preventive effect of EA on cisplatin-induced CIPN was inhibited by downregulation of neuronal IGF1R using AAV vector delivering IGF1R shRNA in this study. Furthermore, downregulation of spinal neuronal IGF1R also prevented the regulating effect of EA on spinal GRK2 in cisplatin-treated mice. The present study indicated that the spinal IGF1 may contribute to the GRK2 role in EA preventing cisplatin-induced CIPN through neuronal IGF1R.

Acupuncture, as one of the commonest therapies in traditional Chinese medicine, has been widely used in clinic for pain management [22–25]. The preclinical researches have proved that acupuncture may activate specific somatosensory neuron, drive the vagal-adrenal axis or specific sympathetic pathways, and restore the physiological functions [26–28]. It plays an important role in regulating glial activation and neuroinflammation [26], improving neuronal dysfunction [29], and controlling peripheral inflammation [27, 28, 30]. Our previous studies have also demonstrated that EA treatment prevented the microglia activation and neuroinflammation in the spinal cord, which had been proved to be involved in cisplatin-induced CIPN in mice, and that EA treatment improved cisplatin-induced peripheral sensory dysfunction [9, 10].

IGF1 has been demonstrated to regulate receptor autophosphorylation and the activity of receptor through extracellular IGF1R [20, 21]. Previous studies have shown that exogenous IGF1 can attenuate oxaliplatin-induced CIPN by reducing the upregulation of IL-17A and TNF-α in the spinal cord [15]. Furthermore, in a rat model of spinal nerve injury, activation of the IGF1/IGF1R pathway has been reported to reverse the elevated expression of proinflammatory cytokines IL-1β, IL-6, and TNF-α [31]. Previous studies reported that depletion of CD11c⁺ microglia or inhibition of IGF1/IGF1R signaling reinstated pain hypersensitivity in peripheral nerve injury models [32]. In diabetic peripheral neuropathy mice, reduced spinal microglial IGF1 expression was observed, while intrathecal IGF1 administration attenuated M1 microglial polarization and suppressed pro-inflammatory cytokine release [33]. These findings emphasize the crucial role of IGF1/IGF1R signaling in regulating microglial activation and neuroinflammation, thereby contributing to pain relief. Taken together, this evidence further supports our results that neuronal IGF1/IGF1R may serve as a critical mediator in EA’s neuroprotective effects against cisplatin-induced CIPN. In this study, EA treatment significantly increased the *Igf1* mRNA and p-IGF1R in the spinal cord. Furthermore, the preventive effect of EA on cisplatin-induced mechanical allodynia, sensory deficit, and microglia activation and neuroinflammation could be inhibited by silencing neuronal IGF1R in the spinal cord. Consistent with our results, the antinociceptive action of IGF1 in the central and peripheral nervous system has also been proved in rats with peripheral tissue injury, and in mice with oxaliplatin-induced peripheral neuropathy [15, 16, 34]. In addition, in a rat model of partial dorsal root ganglionectomies, IGF‑1 has been demonstrated to play a crucial role in EA‑induced neuroplasticity following adjacent dorsal root ganglionectomies via the PI3K/Akt signaling pathway [35]. Therefore, our data suggested that IGF1 in the spinal dorsal horn may exert the antinociceptive or neuroprotective action through neuronal IGF1R, hence giving rise to the preventive process on CIPN by EA.

GRK2 is well known as an important regulatory factor involved in regulating transition from acute pain to chronic pain, and widely distributed in the nervous system [36–38]. Our previous study has showed that overexpression of neuronal GRK2 could prevent cisplatin-induced mechanical allodynia, sensory deficit, and microglia activation and neuroinflammation [9]. EA treatment could prevent the decrease of spinal GRK2 level induced by cisplatin, and the EA effect can be inhibited by either downregulation of neuronal IGF1R or downregulation of neuronal GRK2 in the spinal dorsal horn [9]. In addition, the vital role of neuronal GRK2 in EA regulatory effect on pain and microglia activation has also been proved in a mice model of CFA-induced inflammatory pain [18]. In this study, we further showed that the regulatory effect of EA on GRK2 could be inhibited by downregulation of neuronal IGF1R. Taken together, our study suggested that the spinal IGF1 may regulate neuronal GRK2 by binding neuronal IGF1R, and thus participate in the neuroprotective effect of EA on cisplatin-induced CIPN.

## Conclusions

In summary, the present study showed that EA could upregulate the IGF1/p-IGF1R level in the spinal dorsal horn reduced by cisplatin chemotherapy. The neuroprotective process of EA on cisplatin-induced mechanical allodynia, sensory impairment, and microglia activation and neuroinflammation in the spinal cord was mediated by neuronal IGF1R. Finally, we demonstrated that the neuronal IGF1R mediated the regulatory effect of EA on GRK2 in the spinal dorsal horn. Therefore, we propose that EA may upregulate IGF1, and further regulate GRK2 through neuronal IGF1R in the spinal cord, thus resulting in neuroprotective process on CIPN. This may provide new experimental evidence for the clinical application of acupuncture in the prevention of CIPN.

## Data Availability

No datasets were generated or analysed during the current study.

## Abbreviations

AAV: Adeno-associated virus
ASCO: American Society of clinical oncology
Cd16: Cluster of differentiation 16
Cd206: Cluster of differentiation 206
CIPN: Chemotherapy-induced peripheral neuropathy
Cre: Cyclization recombination enzyme
EA: Electroacupuncture
eGFP: Enhanced green fluorescent protein
ERT: Estrogen receptor
GFAP: Glial fibrillary acidic protein
GRK2: G protein-coupled receptor kinase 2
hSyn: Human synapsin promotor
Iba-1: Ionized calcium-binding adapter molecule-1
Igf1: Insulin-like growth factor 1
IGF1R: Insulin-like growth factor 1 receptor
Il-1β: Interleukin-1 β
Il-4: Interleukin-4
Il-6: Interleukin-6
Il-10: Interleukin-10
NeuN: Neuronal nuclei
inos: Inducible nitric oxide synthase
mA: Milliampere
p-IGF1R: Phosphorated IGF1 receptor
qRT-PCR: Quantitative real-time polymerase chain reaction
shIGF1R: Insulin-like growth factor 1 receptor short hairpin ribonucleic acid
Tnf-α: Tumor necrosis factor
